# Interleukin-1β-Targeted Vaccine Improves Glucose Control and β-Cell Function in a Diabetic KK-A^y^ Mouse Model

**DOI:** 10.1371/journal.pone.0154298

**Published:** 2016-05-06

**Authors:** Jun Zha, Xiao-wei Chi, Xiao-lin Yu, Xiang-meng Liu, Dong-qun Liu, Jie Zhu, Hui Ji, Rui-tian Liu

**Affiliations:** 1 National Key Laboratory of Biochemical Engineering, Institute of Process Engineering, Chinese Academy of Sciences, Beijing, China; 2 School of Pharmacy, China Pharmaceutical University, Nanjing, China; 3 Weifang Biomedical Innovation and Entrepreneurship Service Center, Weifang, China; 4 School of Bioengineering, Qilu University of Technology, Jinan, China; University of Bremen, GERMANY

## Abstract

Interleukin-1β (IL-1β) has been implicated as a key proinflammatory cytokine involved in the pancreatic islet inflammation of type 2 diabetes mellitus (T2DM). Excess IL-1β impairs islet function by inducing insulin resistance and β-cell apoptosis. Therefore, specifically reducing IL-1β activity provides a therapeutic improvement for T2DM by sustaining the inhibition of IL-1β-mediated islet inflammation. In this study, we developed an IL-1β-targeted epitope peptide vaccine adjuvanted with polylactic acid microparticles (1βEPP) and applied it to a diabetic KK-A^y^ mouse model. Results showed that the 1βEPP elicited high antibody responses, which neutralized the biological activity of IL-1β, and induced barely detectable inflammatory activity. 1βEPP immunization reduced body weight gain, protected KK-A^y^ mice from hyperglycemia, improved glucose tolerance and insulin sensitivity, and decreased the serum levels of free fatty acids, total cholesterol and triglyceride. Moreover, 1βEPP restored β-cell mass; inhibited β-cell apoptosis; decreased the expression of IL-1β; and interrupted NF-κB activation by reducing IKKβ and pRelA levels. These studies indicated that the IL-1β-targeted vaccine may be a promising immunotherapeutic for T2DM treatment.

## Introduction

Type 2 diabetes mellitus (T2DM) is a metabolic disorder characterized by continuous deterioration of the insulin secretory capacity leading to insulin resistance and pancreatic β-cells apoptosis [1]. Multiple mechanisms, including glucotoxicity, lipotoxicity, oxidative stress, endoplasmic reticulum stress, and amyloid deposits in the islets [2–4], are involved in the defective insulin secretion and β-cell dysfunction in T2DM, all of these mechanisms are strongly associated with inflammation. Endotoxins, free fatty acids (FFAs), and other lipids recruit fetuin-A, which activates Toll-like receptor 2 (TLR2) and Toll-like receptor 4 (TLR4), thereby leading to the translocation of nuclear factor-κB (NF-κB) (NF-κB) and the release of inflammatory cytokines such as Interleukin-1β (IL-1β), tumor necrosis factor (TNF)-α, and IL-6. These cytokines then activate multiple immune cells and promote the cycling of IL-1β autostimulation [5]. Numerous morphological and therapeutic intervention studies have revealed that inflammation plays a key role in the pathogenesis of diabetes and its complications [6]. Therefore, treatments addressing inflammation could be an effective strategy to prevent T2DM development or delay its onset.

Numerous observations and studies in T2DM patients and animal models revealed that IL-1β is a pivotal proinflammatory cytokine involved in T2DM inflammation [5, 7–10]. Elevated glucose level in pancreatic islets increases the metabolic activity of β-cells, induces the generation of reactive oxygen species (ROS), promotes the activation of the NLRP3 inflammasome, and leads to the production of IL-1β [2]. Moreover, high levels of glucose and FFAs simultaneously activate NF-κB and downstream inflammatory signaling pathways, resulting in the upregulation of proinflammatory genes including IL-1β and TNF-α. IL-1β autostimulation further amplifies inflammation, creating a vicious cycle [11]. Excess IL-1β interferes with pancreas β-cell function, directly leading to insulin secretory dysfunction and β-cell apoptosis [12]. In physiological state, IL-1β exerts robust proinflammatory activities as a part of the innate immune response to pathogen infection. However, IL-1β overproduction in disease states may overcome the natural regulatory mechanisms to drive the pathogenesis of inflammatory disorders [13, 14]. This finding suggests that IL-1β-targeted therapeutic intervention is a reasonable strategy for T2DM treatment. Several clinical trials with the recombinant IL-1 receptor antagonist anakinra [7], humanized IL-1β-specific antibodies XOMA 052 [10], canakinumab [15], and LY2189102 [9], and a vaccine comprising full-length IL-1β protein coupled to Qβ-VLP [16] demonstrated that IL-1β blockade exerts beneficial effects on T2DM by improving glycemic control and enhancing insulin secretion and sensitivity in T2DM patients. However, suitable biotechnology drugs targeting IL-1β approved for T2DM treatment are currently unavailable.

The immunogenic peptide used in our vaccine was composed of 30 amino acid-long neutralizing peptide of IL-1β and VQGEESNDK sequence. The latter corresponds to IL-1β 163–171 amino acids residues (or 47–55 in mature sequence), and can mimic the immunostimulatory effects and adjuvant effects of IL-1β in vivo without inflammatory effects and systemically toxic effects [17]. Microspheres formulated with biodegradable polymers such as polylactic acid (PLA) have been extensively investigated as a vaccine adjuvant because of its controlled release characteristics and biocompatibility [18, 19]. Vaccines with HBsAg or H5N1 influenza virus as immunogens and PLA microparticles as adjuvants [20, 21] elicit strong cellular and humoral immune responses. In the present study, we designed a novel IL-1β-targeted vaccine with IL-1β-derived chimeric peptide as the immunogen and polycation-decorated PLA microspheres as the adjuvant, and then investigated the therapeutic efficacy of this vaccine in a diabetic KK-A^y^ mouse model.

## Materials and Methods

### Vaccine preparation

**Fig 1:**
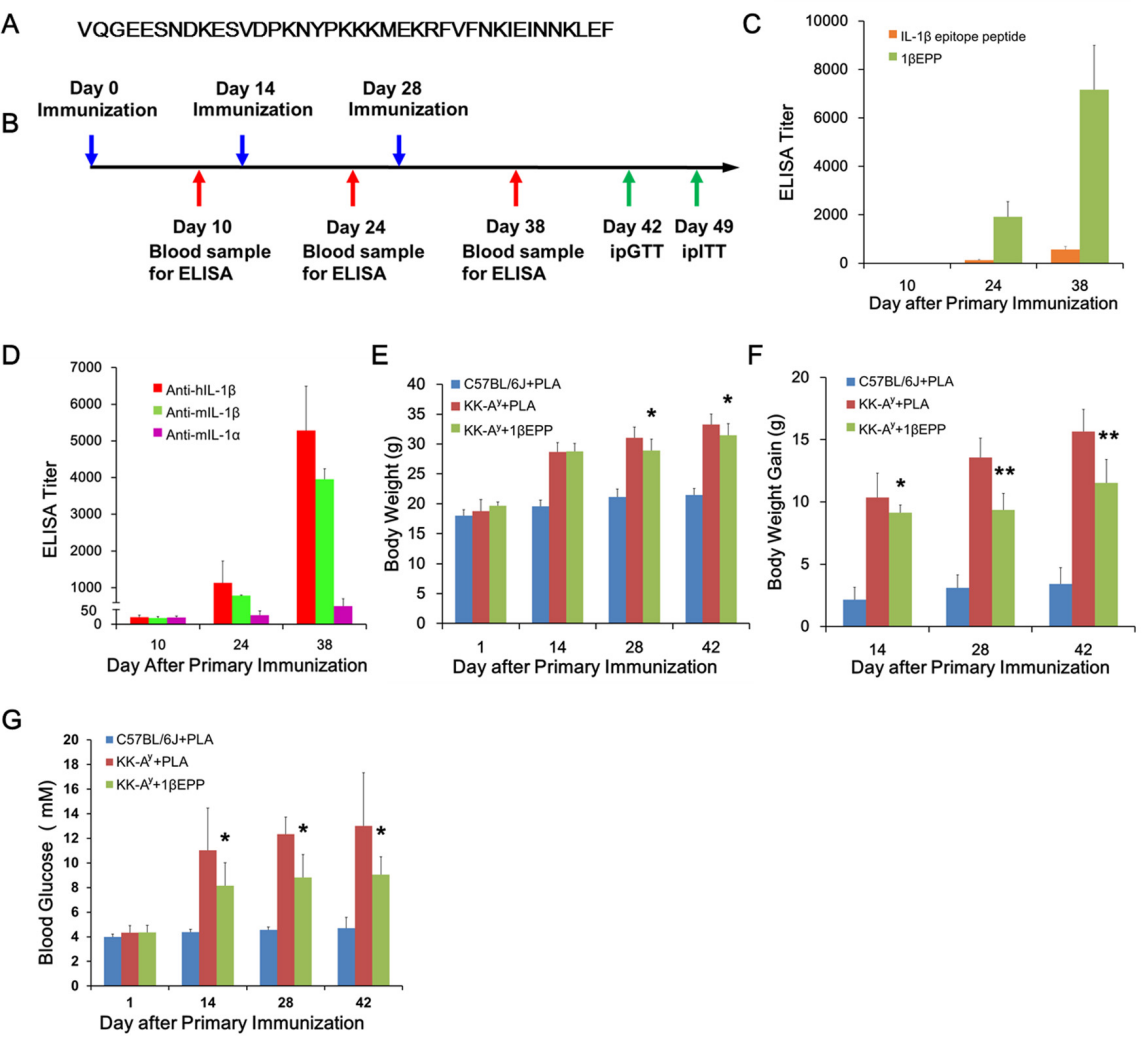
IL-1β targeted vaccine (1βEPP) elicits relatively high antibody responses, decreases body weight gain, and protects from hyperglycemia in KK-A^y^ mice. (A) IL-1β epitope peptide sequence. (B) Work flow and time line of 1βEPP vaccination. (C) ELISA was performed by using IL-1β epitope peptide to detect antibody titers in serum collected from the immunized mice on days 10, 24 and 38 after primary immunization. (D) ELISA was performed by using hIL-1β, mIL-1β or mIL-1α to detect antibody titers. Body weight (E) and body weight gain (F) were measured at the indicated time points. (G) Blood glucose levels were measured at different time points after overnight fasting. Data are shown as mean ± SEM (n = 8). Compared with the KK-A^y^ control mice, *p < 0.05, **p < 0.01.

An epitope peptide containing a 39 amino acid-long epitope of IL-1β (Fig 1A) was synthesized and purified by GL Biochem Co., Ltd. (Shanghai, China). The epitope peptide solution was incubated with an equal volume of PLA microsphere suspension (5 mg/mL in PBS, pH 6.5) in a shaking incubator at 4°C overnight to formulate a PLA microsphere-based vaccine. The solution was centrifuged, and the antigen concentration in the supernatant was analyzed using a micro-bicinchoninic acid protein assay. Antigen adsorption efficiency was calculated as follows [22]:

AE(%)=(mt−ms)÷mt×100

where AE is the adsorption efficiency, mt is the total amount of antigen added to the system, and ms is the amount of antigen in the supernatant.

### Animals and Immunization

Diabetic KK-A^y^ mice have been extensively used as an animal model for T2DM. The mice exhibit symptoms such as an absolute or relative lack of insulin, hyperglycemia, glucose intolerance, and high lipid content, which are similar to the metabolic abnormalities in diabetic patients. Four-week-old male KK-A^y^ mice and male C57BL/6J wild-type mice were obtained from HFK Bioscience Co., Ltd. (Beijing, China). The KK-A^y^ mice were randomly divided into two groups and given a 6% fat diet. KK-A^y^ mice (n = 8) were subcutaneously injected on days 0, 14 and 28 at a 2-week interval with the IL-1β-targeted vaccine adjuvanted with PLA microparticles (1βEPP) containing 50 μg of the epitope peptide. KK-A^y^ control mice and C57BL/6J mice were injected with an equal volume of the PLA adjuvant solution, respectively. Another KK-A^y^ mouse group was injected with an equal volume of the epitope peptide solution for titer determination (Fig 1B). Serum was collected from the tail veins on days 10, 24 and 38 after primary immunization, and antibody titers were determined via ELISA. Body weight and fasting blood glucose concentration were monitored every 2 weeks. To assess if endogenous IL-1β was able to boost 1βEPP-induced antibody titer, KK-A^y^ mice were immunized with 1βEPP. After 14 days, the mice were either i.p. injected with a mixture of 1 ng lipopolysaccharide and 20 mg N-galactosamine or s.c. injected with 50 μg 1βEPP. Antibody titer was measured before injection and 10 days thereafter. When euthanized, the mice were anesthetized with isoflurane and then sacrificed by cervical dislocation. Specific signs used to monitor animal health include: aggression, excessive licking, scratching and rubbing, lethargy, nest building, trembling, bleeding, changes in drinking or eating patterns, swelling and unusual amounts, color or texture of faeces. All mice were regularly anesthetized with isoflurane prior to every procedure to minimize suffering of the mice. All experiments were carried out in accordance with the China Public Health Service Guide for Care and Use of Laboratory Animals. Experiments involving mice and protocols were approved by the Institutional Animal Care and Use Committee of Tsinghua University.

### ELISA

In brief, 96-well ELISA plates (Costar, New York, USA) were coated with 0.5 μg of the IL-1β epitope peptide, human IL-1β (hIL-1β), mouse IL-1β (mIL-1β), or mouse IL-1α (mIL-1α) per well in PBS overnight at 4°C. Plates were blocked by 3% BSA in PBS for 60 min at 37°C. Appropriate dilutions of serum were applied to the plates and then incubated for 60 min at 37°C. The plates were washed and then incubated with horseradish peroxidase-conjugated anti-mouse IgG antibody (Abcam, 1:1000) at 37°C. After 60 min, the plates were washed with PBST again, incubated with 3,3′,5,5′-tetramethylbenzidine (Sigma) for 30 min at room temperature, and then terminated by adding 100 μL of 1 M H_2_SO_4_ per well. The absorbance was measured using an MD-M5 microplate reader at 450 nm. Titers was defined as the highest dilution of serum in the immunized mice with two-fold higher OD value than that in PLA control mice at 450 nm.

### Intraperitoneal glucose tolerance test (ipGTT) and ip insulin tolerance test (ipITT)

For ipGTTs, mice were fasted 12 h and i.p. injected with 40% glucose (Beijing Chemicals, Inc., China) at a dose of 2 g/kg body weight. Blood samples were obtained at time points 0, 15, 30, 60, 90, 120 and 150 min for glucose measurements using a Glucometer (Freestyle; Roche). Serum insulin levels were measured at time points 0 and 30 min by using a mouse insulin-ultrasensitive ELISA kit (ALPCO, Salem, NH). For the ipITT, the mice were i.p. injected with 0.75 U/kg body weight of recombinant human insulin (Novolin; Novo Nordisk, Denmark) after 5 h of fasting, and blood glucose concentration was determined with the Glucometer at time points 0, 15, 30, 60, 90 and 120 min.

### Histochemical analyses

The pancreases of the mice were isolated and fixed in 4% paraformaldehyde at 4°C overnight, followed by paraffin embedding and orienting to make sections cut along the head-tail axis. The mass and apoptosis of β-cells were analyzed as previously described [12]. In brief, a guinea pig anti-insulin primary antibody (Abcam, ab7842) was used followed by detection with goat anti-guinea pig antibody conjugated to Alexa Fluor 488 (Abcam, ab150188) to determine β-cell mass. Images of slides were captured using an Olympus IX73 inverted microscope with a DP80 camera and then analyzed using ImageJ software (National Institutes of Health, Bethesda, MD). β-cell mass was determined by quantifying the cross-sectional β-cell area positive for insulin divided by the cross-sectional area of the total tissue and multiplying this by the pancreatic weight. Apoptosis was analyzed using the terminal deoxynucleotidyl transferase-mediated 2′-deoxyuridine 5′-triphosphate nick-end labeling (TUNEL) technique in accordance with the manufacturer’s instructions (In Situ Cell Death Detection Kit, TMR red; Roche Diagnostics). Subsequently, all sections were triple stained for insulin and 4′,6-diamidino-2-phenylindole (DAPI).

### Homeostatic model of assessment insulin resistance (HOMA-IR)

HOMA-IR was calculated as previously described [23]: fasting insulin (ng/ml) × fasting glucose (mmol/l) = HOMA-IR.

### Quantitative RT-PCR analysis

The islets were isolated as described previously [4]. In brief, the pancreas were perfused with collagenase solution (HBSS supplemented with 25 mM HEPES, 0.5 mg/ml collagenase P, and 0.1 mg/ml DNase), digested in this collagenase solution at 37°C, and then dispersed by shaking for 30 seconds. Islets were manually picked, and washed with ice-cold PBS for subsequent RNA extraction and quantitative RT-PCR analysis. Total RNA was isolated using the RNeasy Lipid Tissue kit (QIAGEN, Inc., Valencia, CA). cDNA was prepared from 1.5 μg of RNA by using a PrimeScript RT-PCR kit (Takara). Quantitative RT-PCR for IL-1β was performed with the 7500 Fast Real-Time PCR System (Life Technologies) and SYBR Select Master Mix (Applied Biosystems) for amplification and detection. The expression of IL-1β was normalized to the expression of GAPDH.

The primers used for quantitative RT-PCR were as follows: 5′-CAACCAACAAGTGATATTCTCCATG-3′ and 5′-GATCCACACTCTCCAGCTGCA-3′ for IL-1β, and 5′-TCCATGACAACTTTGGCATTG-3′ and 5′-CAGTCTTCTGGGTGGCAGTGA-3′ for GAPDH.

### Western blot analysis

The pancreas tissues of KK-A^y^ and C57BL/6J mice were collected and homogenized in 20 mM Tris-HCl (pH 7.4) containing 1% sodium dodecyl sulfide (SDS) and 1:10 of a protease inhibitor cocktail (Sigma). The protein extracts were boiled and separated by 4%-12% NuPAGE gels (Invitrogen) and then transferred onto a nitrocellulose membrane. After blocking with 5% nonfat dry milk in PBST, the membranes were incubated with primary antibodies against pRelA (clone 93H1, 1:1,000), total IKKβ (clone L570, 1:1,000), α-tubulin (#2144, 1:1,000) and GAPDH (clone 14C10, 1:1,000) (Cell Signaling, Beverly, MA), respectively. The membranes were washed thrice in PBST before being incubated in IR secondary antibodies (1:5000; Li-Cor; Lincoln, NE, USA; #926–3211 and #926–68020) for 1 h at room temperature. The membranes were washed thrice again and then imaged in the Li-Cor Odyssey IR detection system. Densitometry was carried out using the integrated intensity value for each band.

### Serum lipid analysis

Blood samples were obtained from mouse tail veins on day 56 after primary immunization. Serum lipid including FFA, total cholesterol (TC), HDL-cholesterol, and triglyceride (TG) was measured using commercial kits (Nanjing Jiancheng Bioengineering Institute, Nanjing, China) in accordance with the manufacturer’s instruction. All lipid measurements were performed in triplicate. Non-HDL cholesterol levels were calculated by subtracting HDL-cholesterol from TC.

### *In vivo* assay for IL-1β activity

Groups of C57BL/6J mice (n = 6) were injected i.p. with 1 μg of either hIL-1β or hIL-1β epitope peptide or s.c. with 50 μg of 1βEPP, vaccine-treated KK-A^y^ mice were received i.p. injection of 1μg hIL-1β. Three hours later, serum IL-6 levels were quantified using an IL-6 quantitative ELISA kit (Neobioscience Technology, Beijing, China).

### Statistical analyses

Data are presented as mean ± SEM and then analyzed by one-way ANOVA with a Newman-Keuls *post-hoc* test and/or a Student’s *t*-test for paired comparisons. Statistical significance was set at p < 0.05.

## Results

### IL-1β targeted vaccine (1βEPP) elicits high antibody responses in mice

The IL-1β-targeted vaccine 1βEPP was prepared by combining the IL-1β epitope antigen with polycation-decorated PLA microsphere-based adjuvants. Our results showed that the antigen adsorption capacity by PLA microspheres was 56%. To assess vaccine efficacy, we immunized KK-A^y^ mice with 1βEPP thrice at a 2-week interval. The antibody titer gradually increased after primary immunization. IgG titers in 1βEPP-treated mice were detectable on day 24 and elevated to around 7,000 on day 38, whereas the IL-1β epitope peptide alone induced low antibody titers (Fig 1C). To investigate if 1βEPP-induced antibodies could recognize IL-1β, we performed ELISA using both hIL-1β and mIL-1β. The results showed that 1βEPP-induced anti-hIL-1β and anti-mIL-1β ELISA titer were 5280 and 3952 after 3-time immunization, respectively, indicating that the IL-1β-targeted vaccine adjuvanted with PLA elicited relatively high antibody responses in KK-A^y^ mice (Fig 1D). Our results also showed that 1βEPP-induced antibody barely bond to mIL-1α, suggesting that the induced-antibodies had minimal cross-reactivity with mIL-1α (Fig 1D).

### IL-1β targeted vaccine (1βEPP) decreases body weight gain in KK-A^y^ mice

To estimate the effects of 1βEPP on T2DM, we applied the vaccine to a KK-A^y^ diabetic mouse model that develops obesity and hyperglycemia with insulin resistance spontaneously. Compared with the C57BL/6J wild-type mice, all KK-A^y^ mice treated with or without the vaccine gained more weight, but 1βEPP treatment significantly decreased the weight gain of KK-A^y^ mice after second immunization (Fig 1E and 1F). The average body weights of the KK-A^y^ control mice and 1βEPP-treated mice were 33.2 and 31.8 g on day 42 after primary immunization, respectively (Fig 1E).

### IL-1β targeted vaccine (1βEPP) protects KK-A^y^ mice from hyperglycemia and improves glucose tolerance and insulin sensitivity

We measured the fasting blood glucose levels of the mice every 2 weeks during the immunization period to evaluate the effects of 1βEPP on glycemic control. The KK-A^y^ mouse control group spontaneously developed hyperglycemia, and the fasting blood glucose levels increased from 4.3 mM to 13 mM within 6 weeks. 1βEPP diminished the increase of the blood glucose in KK-A^y^ mice. The blood glucose level of 1βEPP-treated mice was approximately 8.2 mM after primary immunization, which was significantly lower than that of the KK-A^y^ mouse control group. On days 28 and 42, 1βEPP-treated mice kept remarkably low blood glucose levels (Fig 1G). These data demonstrated that 1βEPP could protect KK-A^y^ diabetic mice from hyperglycemia.

**Fig 2:**
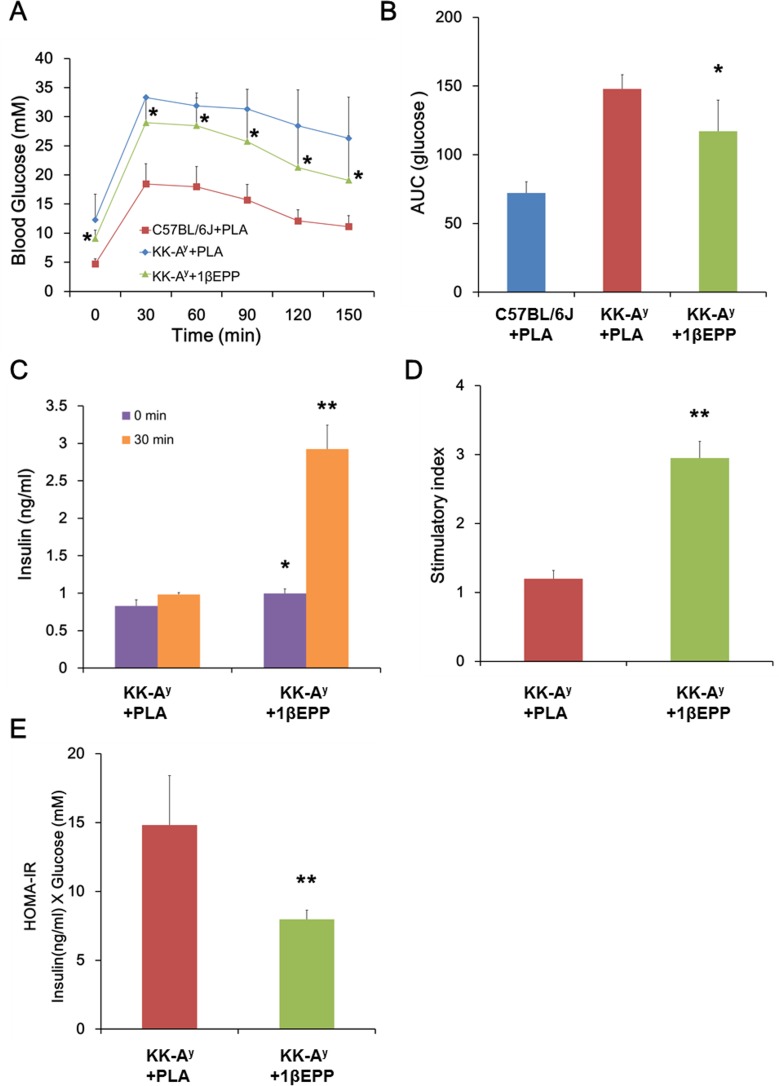
IL-1β targeted vaccine (1βEPP) improves glucose tolerance in KK-A^y^ mice. (A) 1βEPP -treated KK-A^y^ mice were challenged with 2 g/kg body weight of glucose, and then blood glucose levels were measured at 0, 30, 60, 90, 120 and 150 min. (B) Area under the curve (AUC) for blood glucose (0–150 min) during the ipGTT was calculated. (C) Serum insulin was measured during the ipGTT at 0 and 30 min after glucose injection. (D) Insulin stimulatory index as a ratio of stimulated (30 min) over basal (0 min) serum insulin concentration. (E) HOMA-IR as a product of fasting insulin and blood glucose levels. Data are shown as mean ± SEM (n = 8). Compared with the KK-A^y^ control mice, *p < 0.05, **p < 0.01.

We performed ipGTT on day 42 after the first vaccine immunization to test the effects of 1βEPP on glucose tolerance. The fasted mice were challenged with 2 g/kg body weight of glucose, and the blood glucose levels were determined every 30 min after glucose injection. The KK-A^y^ control mice displayed impaired glucose tolerance, resulting in markedly high glucose levels at 0, 30, 60, 90, 120 and 150 min, whereas 1βEPP treatment improved the glucose control with low blood glucose levels at all time points (Fig 2A). Area under the curve (AUC) analysis revealed a significantly lower blood glucose concentration in 1βEPP-treated KK-A^y^ mice than in the control mice (Fig 2B). We also assessed insulin secretion at 0 and 30 min in the ipGTT (Fig 2C). The KK-A^y^ control mice could not markedly increase insulin levels in response to glucose challenge, whereas 1βEPP-immunized KK-A^y^ mice restored insulin secretion with glucose stimulation (Fig 2D). HOMA-IR was analyzed as the product of fasting insulin and fasting blood glucose. The HOMA-IR value significantly decreased in 1βEPP-treated mice compared with the KK-A^y^ control mice (Fig 2E). These results indicated that 1βEPP significantly improved glucose tolerance in KK-A^y^ mice.

**Fig 3:**
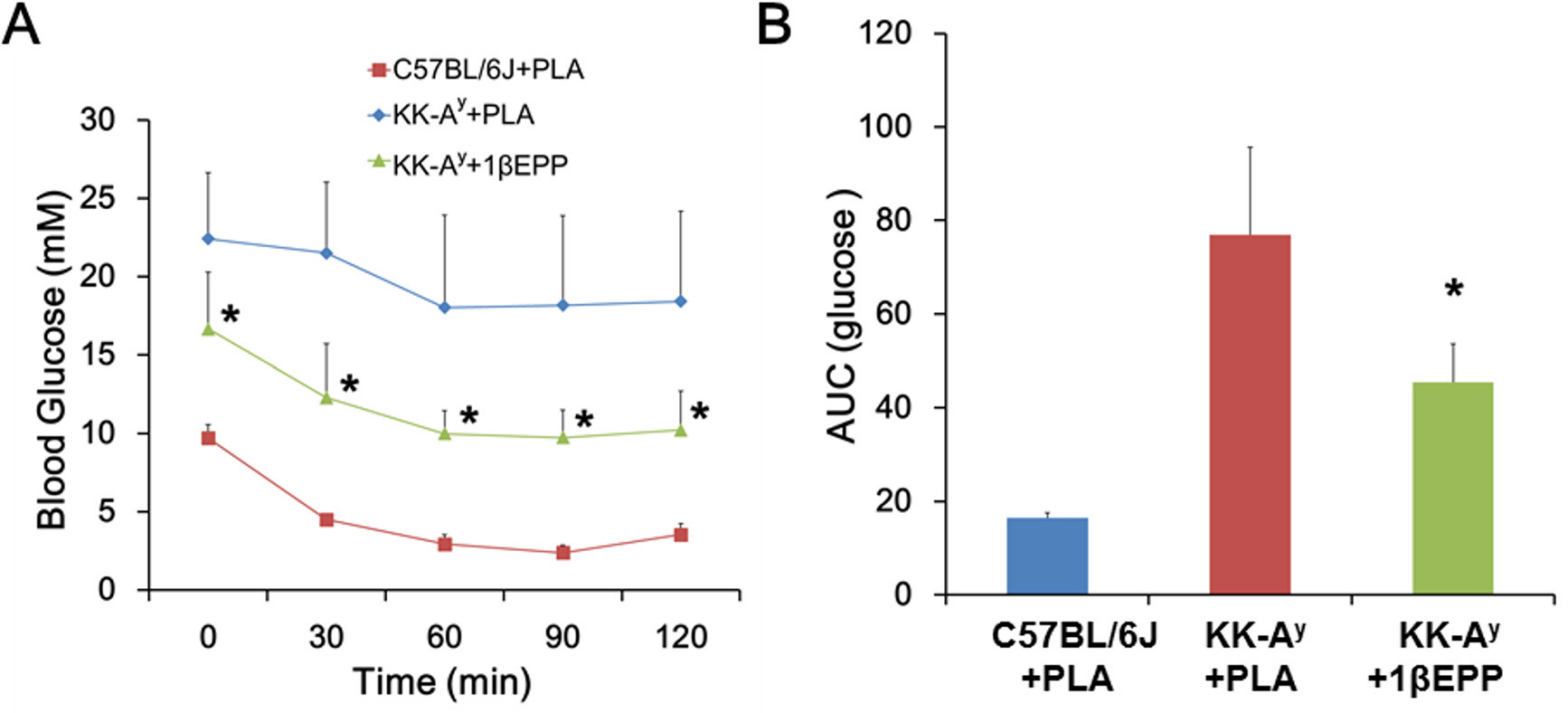
IL-1β targeted vaccine (1βEPP) improves insulin secretion and insulin sensitivity in KK-A^y^ mice. (A) KK-A^y^ mice were fasted for 5 h before ipITT was performed. Blood glucose levels were measured at 0, 30, 60, 90 and 120 min after i.p. injection of insulin (0.75 U/kg body weight). (B) AUC for blood glucose (0–120 min) was calculated during the ipITT. Data are shown as mean ± SEM (n = 8). Compared with the KK-A^y^ control mice, *p < 0.05.

We conducted ipITT after the third vaccine immunization to assess the effects of 1βEPP on insulin sensitivity. Blood glucose levels were measured every 30 min after a single i.p. injection of insulin (0.75 U/kg body weight). The KK-A^y^ control mice showed impaired insulin sensitivity with markedly higher blood glucose levels than the C57BL/6J mice, whereas 1βEPP alleviated the insulin resistance and decreased the blood glucose levels in the KK-A^y^ mice (Fig 3A). AUC analysis also showed that the blood glucose levels of 1βEPP-treated mice were much lower than those of the control mice (Fig 3B).

### IL-1β targeted vaccine (1βEPP) restores β-cell mass and protects KK-A^y^ mice from β-cell apoptosis

**Fig 4:**
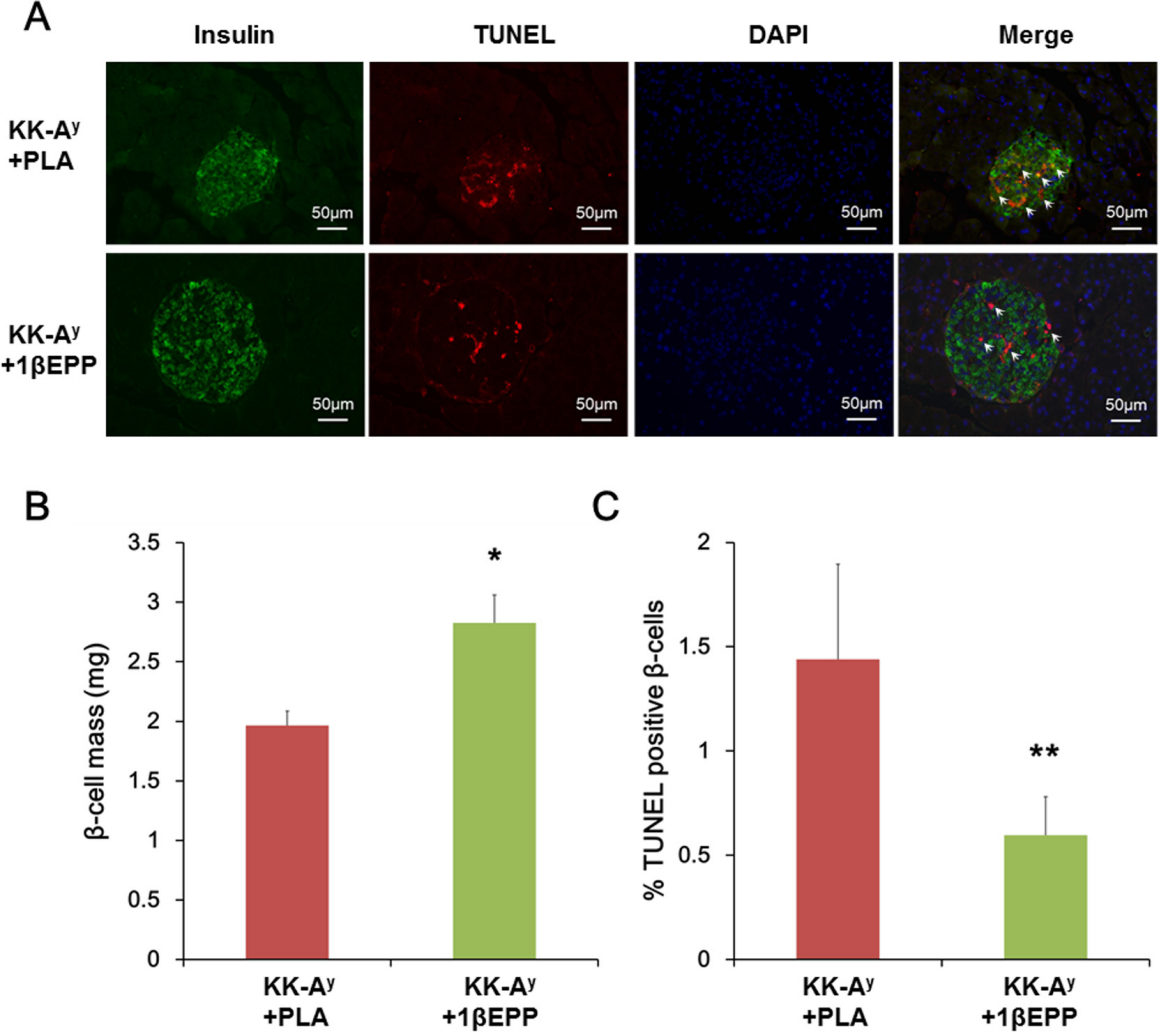
IL-1β targeted vaccine (1βEPP) restores β-cell mass and protects from β-cell apoptosis. (A) Triple staining for terminal deoxynucleotidyl transferase dUTP nick end labeling (TUNEL) (*red*), insulin (*green*), and DAPI (*blue*) was performed on fixed, paraffin-embedded pancreas sections. (B) β-cell mass per pancreas was estimated as the relative cross-sectional area of β-cells (determined by quantifying the cross-sectional area occupied by β-cells divided by the cross-sectional area of total tissue) multiplied by the weight of the pancreas. (C) β-cell apoptosis was quantified by percentage of TUNEL-positive β-cells. Ten sections from each pancreas spanning the width of the pancreas were included in the analysis. Data are shown as mean ± SEM. Compared with the KK-A^y^ control mice, *p < 0.05, **p < 0.01.

The prominent pathological characteristics of T2DM are insulin resistance, β-cell dysfunction, and β-cell mass decline. We performed histochemical analysis on pancreas sections to investigate whether 1βEPP affects β-cell mass. 1βEPP restored β-cell mass by an increase of 44% compared with the KK-A^y^ control mice (Fig 4A and 4B). We detected β-cell apoptosis to estimate the effects of 1βEPP on β-cell survival. The results of immunohistochemical staining revealed that the amount of TUNEL-positive β-cells was markedly higher in the pancreas sections of the KK-A^y^ control mice than in those of 1βEPP-treated mice (Fig 4A). Compared with the KK-A^y^ control mice, 1βEPP-treated mice had 59% lower β-cell apoptosis (Fig 4C). These results indicated that 1βEPP restored β-cell survival by reducing apoptosis.

### IL-1β targeted vaccine (1βEPP) reduces IL-1β gene expression in KK-A^y^ mice

**Fig 5:**
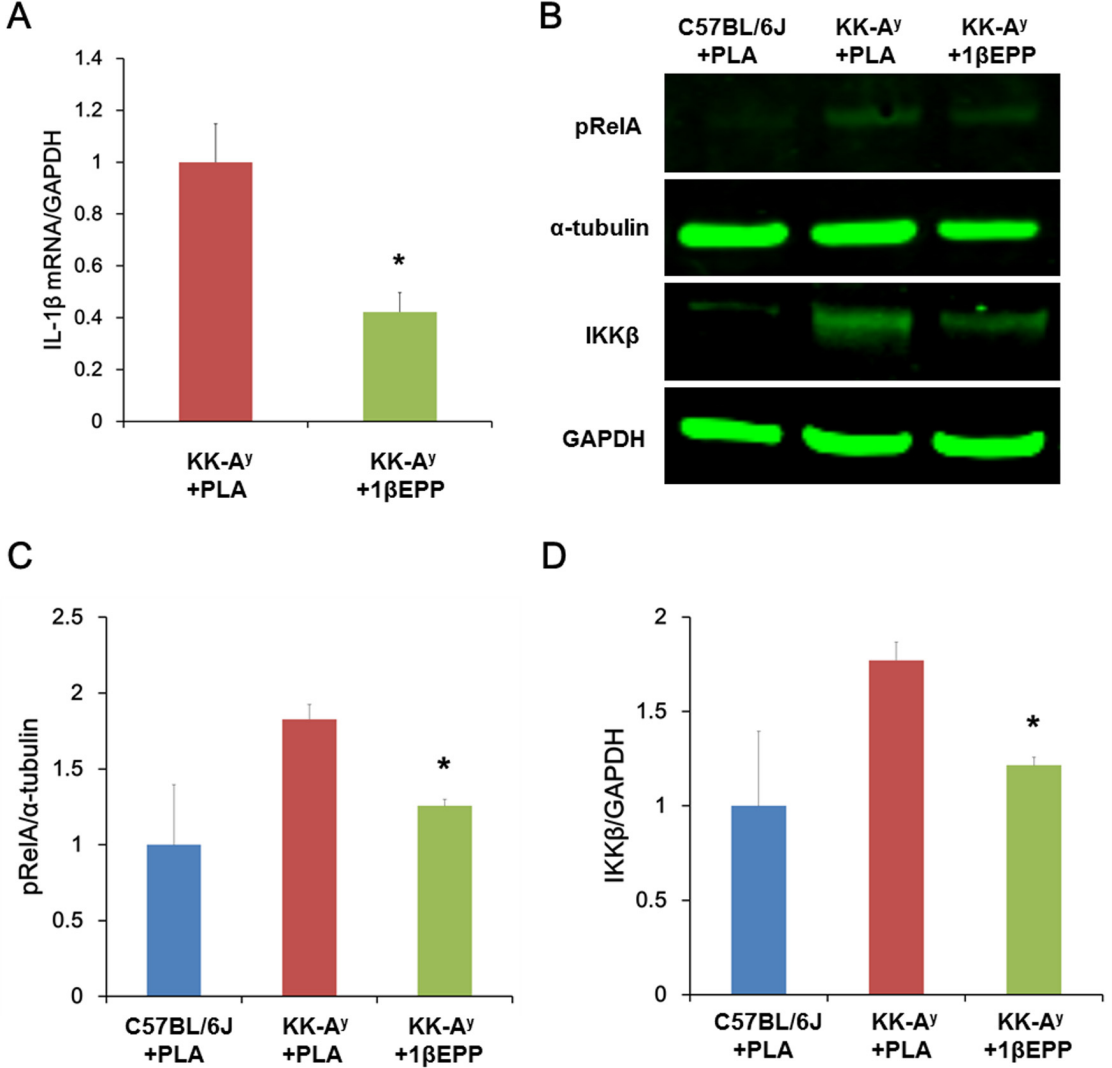
IL-1β targeted vaccine (1βEPP) reduces IL-1β gene expression and inhibits NF-κB activation in KK-A^y^ mice. (A) mRNA levels of IL-1β in pancreas islets were measured by quantitative RT-PCR after thrice of 1βEPP vaccination. (B) Phosphorylated RelA (pRelA) (α-tubulin as a loading control) and total IKKβ (GAPDH as a loading control) levels in pancreas tissues of KK-A^y^ mice were detected by Western blot. Intensities of pRelA (C) and IKKβ (D) bands were quantified using ImageJ software and then normalized to α-tubulin or GAPDH, respectively. Data are shown as mean ± SEM (n = 8). Compared with the KK-A^y^ control mice, *p < 0.05.

We evaluated the mRNA levels of IL-1β to determine whether the improved glucose tolerance and insulin sensitivity correlate with the reduced expression of IL-1β. The results showed 1βEPP significantly reduced the IL-1β mRNA levels in islets of KK-A^y^ mice by 57.8% (Fig 5A), suggesting that 1βEPP could decrease pancreas inflammation in the KK-A^y^ mice by inhibiting IL-1β gene expression.

### IL-1β targeted vaccine (1βEPP) inhibits NF-κB activation in KK-A^y^ mice

Activation of NF-κB and downstream inflammatory signaling pathways are key events in the etiology of insulin resistance and β-cell dysfunction. Under resting conditions, NF-κB forms a complex with the inhibitory subunit IκB. NF-κB is released by IκB phosphorylation and proteasome-mediated degradation and translocated into the nucleus, where it promotes the transcription of genes that encode inflammatory mediators [24]. The IKKβ and phosphorylation of RelA (pRelA) levels in the pancreas homogenate of KK-A^y^ mice were detected by Western blot to investigate whether 1βEPP inhibited NF-κB activation. IKKβ and downstream pRelA levels were significantly lower in 1βEPP-treated KK-A^y^ mice than in the control mice (Fig 5B). Quantification analysis demonstrated that the levels of pRelA (Fig 5C) and IKKβ (Fig 5D) in 1βEPP-treated KK-A^y^ mice decreased by 31.2% and 31.3%, respectively. This result indicated that 1βEPP can inhibit NF-κB activation.

### IL-1β targeted vaccine (1βEPP) reduces serum lipid levels in KK-A^y^ mice

**Fig 6:**
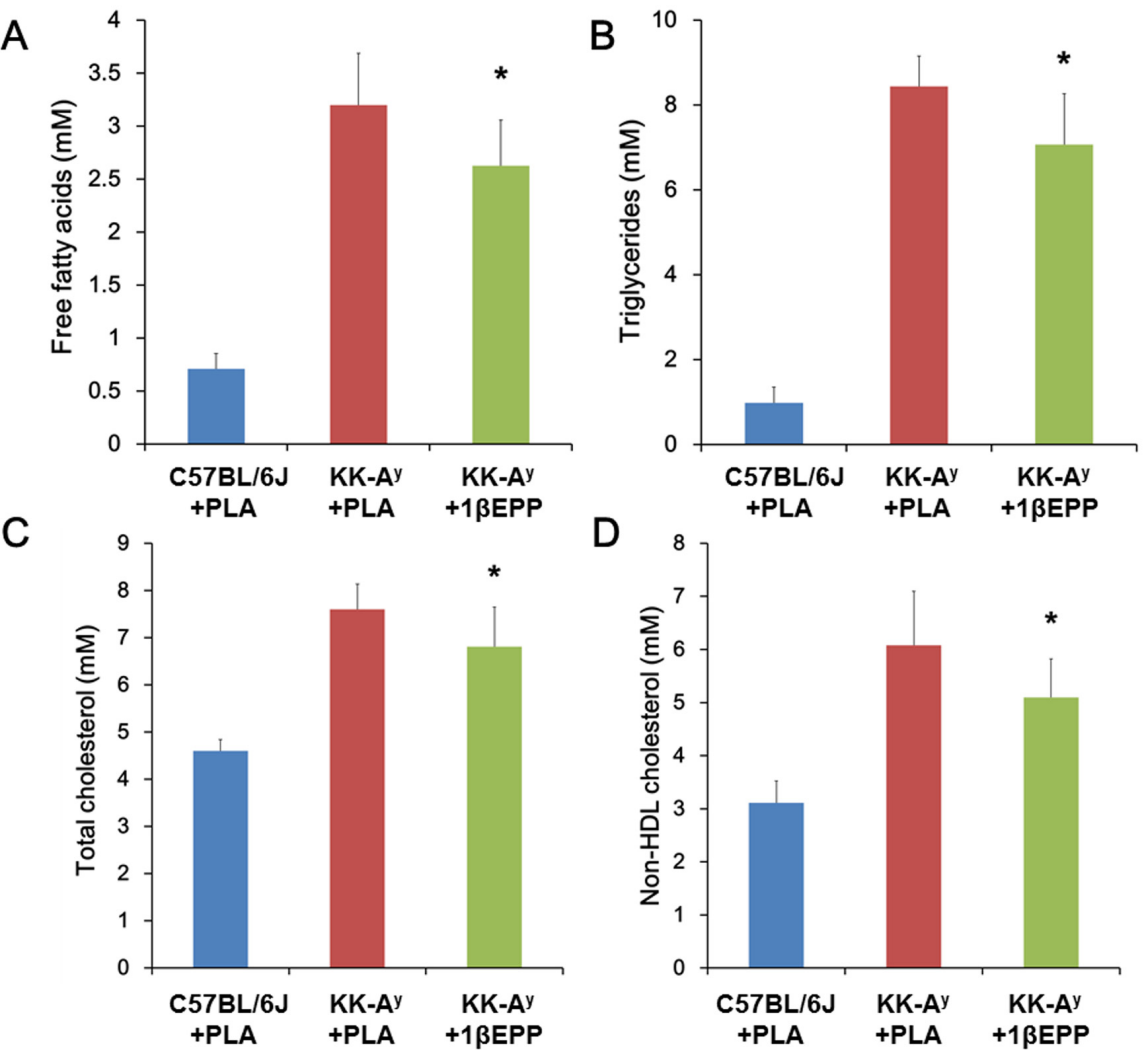
IL-1β targeted vaccine (1βEPP) reduces serum lipid levels in KK-A^y^ mice. Serum levels of free fatty acids (A), triglycerides (B), and total cholesterol (C) were measured on day 56 after the primary immunization. Non-HDL cholesterol (D) was obtained by subtracting HDL-cholesterol from the total cholesterol. Data are shown as mean ± SEM (n = 8). Compared with the KK-A^y^ control mice, *p < 0.05.

Serum lipid levels are strongly associated with insulin secretion and sensitivity. Previous studies demonstrated that the serum levels of FFAs, TC and TGs are significantly higher in adult KK-A^y^ mice than in age-matched C57BL/6J mice [25]. In the present study, 1βEPP significantly reduced the serum levels of FFA, TGs, TC and non-HDL cholesterol in the KK-A^y^ mice by 17.9%, 16.2%, 10.4% and 16.1%, respectively, compared with the control KK-A^y^ mice (Fig 6).

### Bioactivity and safety assessment of 1βEPP

**Fig 7:**
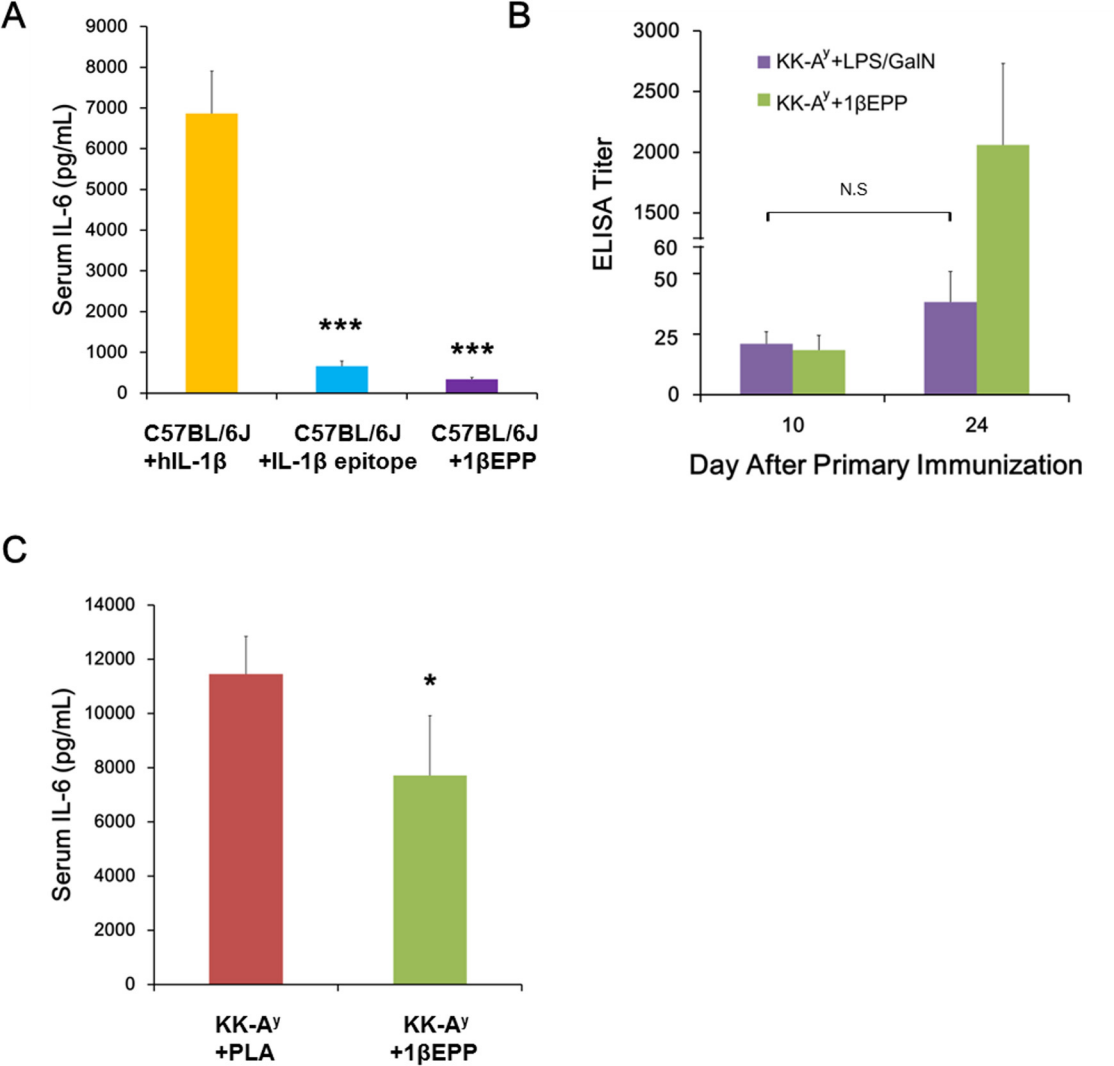
Bioactivity and safety assessment of 1βEPP. (A) *In vivo* inflammatory activity. Groups of C57BL/6J mice were received i.p. injection of 1μg hIL-1β, IL-1β epitope peptide or s.c. injection of 50 μg 1βEPP. Three hours later, serum IL-6 levels were quantified using an IL-6 quantitative ELISA kit. (B) Effects of endogenous IL-1β on antibody titer. KK-A^y^ mice were immunized once with 1βEPP and subsequently i.p. injected with 1 ng lipopolysaccharide and 20 mg N-galactosamine or s.c. injected with 50 μg 1βEPP. Antibody titer was measured before injection and 10 days thereafter. (C) *In vivo* neutralization activity to IL-1β. After immunized with either 1βEPP or PLA for three times, KK-A^y^ mice were received i.p. injection of 1 μg hIL-1β, three hours later, serum IL-6 levels were quantified using an IL-6 quantitative ELISA kit. Data are shown as mean ± SEM (n = 6). *p < 0.05, ***p < 0.001.

To compare the safety of the hIL-1β epitope peptide with IL-1β *in vivo*, Groups of C57BL/6 mice (n = 6) were injected i.p. with 1 μg of either hIL-1β or hIL-1β epitope peptide or s.c. with 50 μg of 1βEPP. The results showed that the injection of both hIL-1β epitope peptide and 1βEPP induced significantly lower serum concentrations of IL-6 compared with hIL-1β (Fig 7A). Thus, 1βEPP may avoid or reduce acute IL-1β-related toxicity induced by full-length IL-1β vaccine injection.

To assess if endogenous IL-1β was able to boost 1βEPP-induced antibody titer, mice were immunized once with 1βEPP and subsequently injected with lipopolysaccharide and N-galactosamine in order to induce the endogenous increase of IL-1β. Antibody titer was measured before injection and 10 days thereafter. Results showed that lipopolysaccharide and N-galactosamine injection had no significant influence on antibody titers, indicating that increased levels of endogenous IL-1β did not boost the existing antibody response induced by 1βEPP and only an additional injection of 1βEPP could boost the antibody response (Fig 7B).

To determine if 1βEPP -induced antibody was able to neutralize the biological activity of IL-1β, we performed the challenge experiments *in vivo*. Our results showed that the IL-1β challenge induced serum IL-6 level to around 11,000 pg/mL in KK-A^y^ control mice, while 1βEPP inhibited the challenge effect by 32.7%, to 7,700 pg/mL (Fig 7C).

## Discussion

T2DM manifests as a result of impaired β-cell function, decreased β-cell mass and insulin resistance [23]. Recent evidence has suggested that IL-1β plays a critical role in T2DM and that inhibiting IL-1β can improve glucose control and β-cell function [26]. In present study, we designed a novel peptide vaccine specially targeting IL-1β to improve glycemic control and insulin sensitivity, decrease lipid profile and β-cell apoptosis.

Vaccines have been the most relevant therapy for the prevention of infectious diseases. Recently, therapeutic vaccines targeting molecular risk factors associated with chronic diseases have been developed successfully, examples of these vaccines include those against angiotensin II and nicotine [27]. Compared with antibodies, vaccines are a cheaper therapy because of their long-lasting effects and lack of daily dosage requirements. Previous studies reported that active immunization by the peptide regions of mIL-1β resulted in significant high levels of autoantibodies against the native mIL-1β and showed protective effects against inflammation and collagen-induced arthritis in mice [28], indicating that the IL-1β-targeted vaccine may provide these advantages to treat T2DM patients. However, phase I/IIa trials with the VLP-based vaccine IL-1bQb containing full-length IL-1β in T2DM patients obtained unacceptable side effects. To develop an ideal therapeutic vaccine for T2DM, Spohn G et al. recently developed a genetically detoxified form of hIL-1β as vaccine antigen, which carried an N-terminal extension of three amino acids (Met-Asp-Ile), and an amino acid substitution from aspartate to lysine at position 145. The VLP-based vaccine containing this mutant form of IL-1β showed preferable safety and efficacy in mice [16]. In our study, a 39 amino acid-long neutralizing epitope from IL-1β was used as the immunogen, which partly maintained the IL-1β immunogenicity and considerably avoided or reduced vaccine-related acute toxicity.

The induction of high antibody titers against both hIL-1β and mIL-1β by 1βEPP in mice indicated that the selected epitope peptide had strong immunogenicity. 1βEPP significantly decreased body weight gain in KK-A^y^ diabetic mice and improved glucose tolerance during the ipGTT. This result is consistent with the strong association between glucose tolerance and body weight in KK-A^y^ diabetic mice [25]. The present study also indicated that 1βEPP decreased hyperglycemia in KK-A^y^ mice. The blood glucose levels in 1βEPP-treated mice were obviously lower than those in the KK-A^y^ control mice at all time points after glucose challenge. AUC analysis also confirmed the beneficial effects of 1βEPP in maintaining glucose homeostasis. These effects of 1βEPP possibly resulted from the improved insulin secretion and sensitivity. During the ipGTT, the serum insulin levels significantly increased in 1βEPP-immunized KK-A^y^ mice compared with the KK-A^y^ control mice, which improved glucose control. Moreover, insulin sensitivity was significantly improved by 1βEPP treatment as assessed via the ipITT and the HOMA-IR index. These results indicated that 1βEPP offered protective effects on insulin sensitivity and glucose control.

Elevated glucose levels in T2DM impair β-cell function and induce apoptosis, accelerating the progression of T2DM [23]. IL-1β is a key mediator of β-cell dysfunction and apoptosis, excess IL-1β impairs the compensatory capacity of β-cells [3] and induces a vicious cycle through its autostimulation. In the present study, 1βEPP significantly increased β-cell mass and decreased β-cell apoptosis in the KK-A^y^ mice. These protective effects of 1βEPP possibly contributed to its ability to inhibit IL-1β production. Further studies using quantitative RT-PCR analysis confirmed that 1βEPP reduced IL-1β transcription in KK-A^y^ mice. Inflammation is also an important pathogenic pathway in the development of diabetic complications [29]. NF-κB activation is an initial and crucial step in T2DM evolution that contributes to both insulin resistance and β-cell apoptosis, and provides a link between metabolic inflammation and obesity development [30]. Our data indicated that 1βEPP inhibited NF-κB activation by reducing IKKβ and pRelA levels, and thus blocked the downstream inflammatory signaling pathway and downregulated the expression of IL-1β.

High serum lipids such as FFA, TC and TG directly mediated insulin resistance and induced β-cell apoptosis, which led to T2DM and increased the cardiovascular risks [23]. 1βEPP lowered FFA, TC and TGs, which may further attenuate insulin resistance and β-cell apoptosis. Due to the strong pyrogenic and hypotensive activity of wide-type IL-1β that might lead to fever and hypotension in vaccinated patients, it is very important to develop a safe and effective IL-1β-targeted vaccine without these side effects of full-length IL-1β. 1βEPP induced barely detectable inflammatory activity and showed strong ability to neutralize IL-1β biological activity *in vivo*. Moreover, 1βEPP-induced antibody titers were not boosted by endogenous IL-1β. In conclusion, the present work provided an evidence in a T2DM mouse model that IL-1β blockage is effective in treating T2DM, further suggesting that active immunization targeting IL-1β may be a potential therapeutic for T2DM treatment. Several conditions driven by inflammatory processes are also associated with rheumatoid arthritis, gout, psoriasis, and Crohn’s disease. Therefore, 1βEPP may act on a dysfunctional pathway that causes several conditions associated with some metabolic syndrome disorders aside from T2DM. However, the interruption of the vicious cycles of IL-1β autoinduction and the long-lasting effects of the vaccine suggest that the optimal dose and treatment duration need to be carefully considered in future studies.

## Abbreviations

T2DM: Type 2 diabetes mellitus
IL-1β: interleukin-1β
1βEPP: IL-1β-targeted epitope peptide vaccine adjuvanted with polylactic acid microparticles
NF-κB: nuclear factor-κB
